# Supplementary material for: Dendrobium alkaloids decrease Aβ by regulating α- and β-secretases in hippocampal neurons of SD rats
Source: PeerJ. 2019 Sep 6;7:e7627. doi: 10.7717/peerj.7627 (PMC6733236; doi:10.7717/peerj.7627)

**Full-length uncropped blots**

**Fig. 4. *DNLA decreased the accumulation of Aβ through decreasing APP***

(A)the protein expression of Aβ_1-42_

Aβ_1-42_ control 0.35 3.5 35 350 700ng/mL


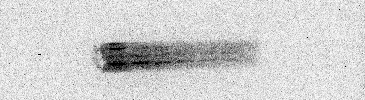

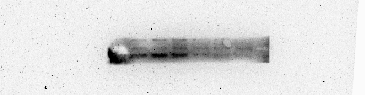


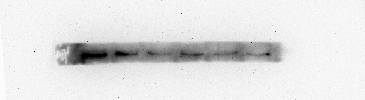


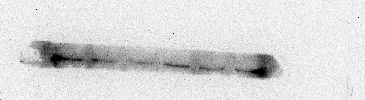


β-ACTIN


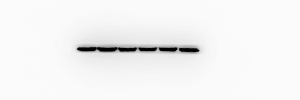


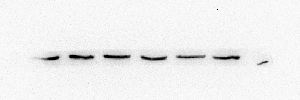


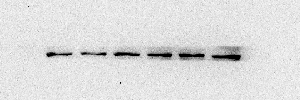


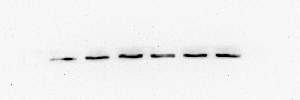


(B)the protein expression of PS1

PS1 control 0.35 3.5 35 350 700ng/mL


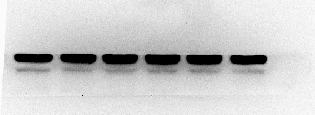


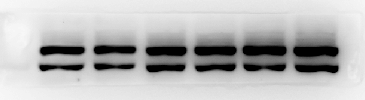


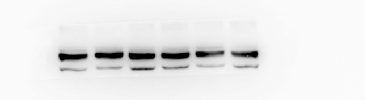


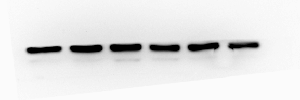


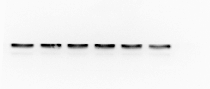


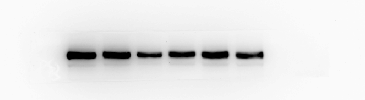


GAPDH


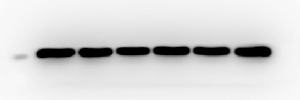


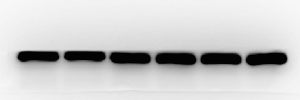


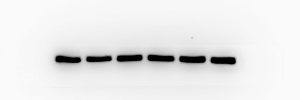


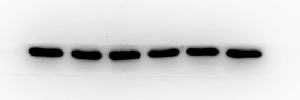


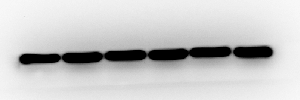


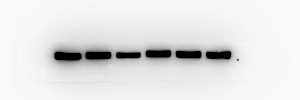


he protein expression of APP

(C) APP control 0.35 3.5 35 350 700ng/mL


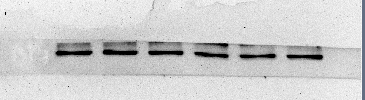


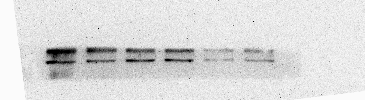


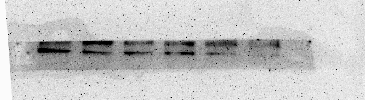


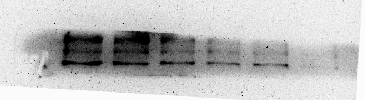


GAPDH


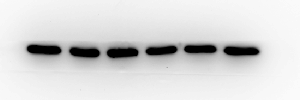


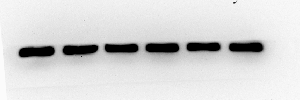


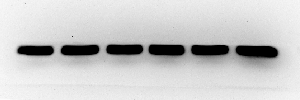


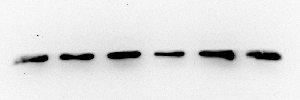


**Fig. 5.** DNLA reduced the production of Aβ through non-amyloidogenic pathways

1. the protein expression of ADAM10

ADAM10 control 0.35 3.5 35 350 700ng/mL


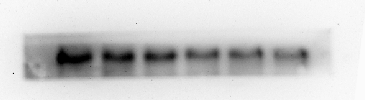

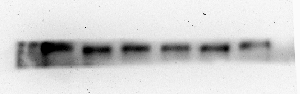


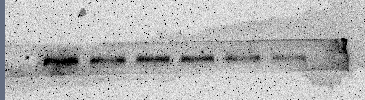
4


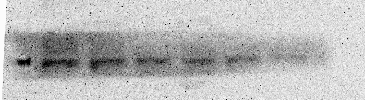


GAPDH


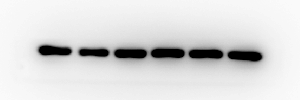


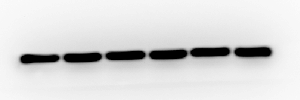


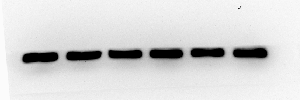
4


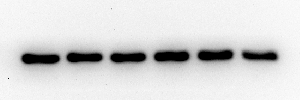


1. the protein expression of ADAM17

ADAM17 control 0.35 3.5 35 350 700ng/mL


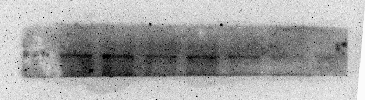


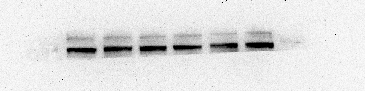


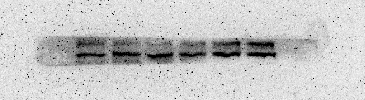


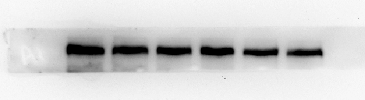


GAPDH


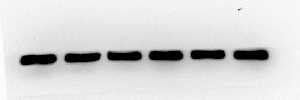


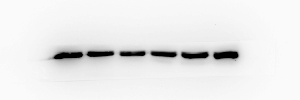


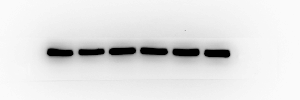


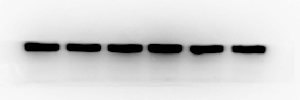


**Fig. 6. *DNLA decreased accumulation of Aβ by inducing BACE1 (β-secretase)***

the protein expression of ***BACE1***

***BACE1*** control 0.35 3.5 35 350 700ng/mL


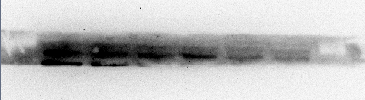


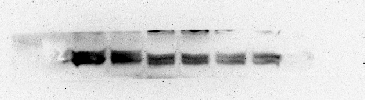


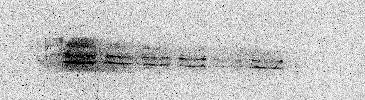


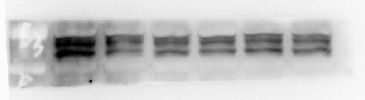


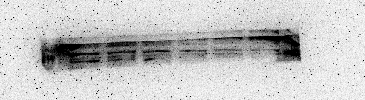


GAPDH


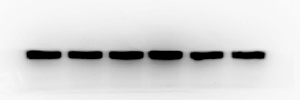


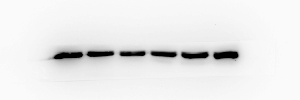


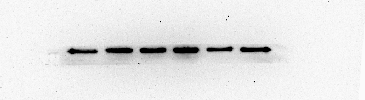


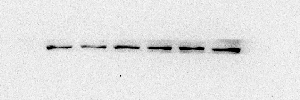


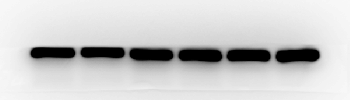

Supplement: Dataset S2 [file peerj-07-7627-s002.docx]
